# Randomized controlled trials of pharmacological treatments to prevent COPD exacerbations: applicability to real-life patients

**DOI:** 10.1186/s12890-019-0882-y

**Published:** 2019-07-12

**Authors:** Laurie Pahus, Pierre-Régis Burgel, Nicolas Roche, Jean-Louis Paillasseur, Pascal Chanez, on behalf of Initiatives BPCO scientific committee

**Affiliations:** 1 Clinique des bronches allergies et sommeil, Aix Marseille Univ, APHM, CIC 9502, 13015 Marseille, France; 2 ADES, Aix Marseille Univ, CNRS, EFS, Marseille, France; 3 C2VN (INSERM U1263, INRA 1260), Aix Marseille Univ, Marseille, France; 4 Service de Pneumologie, GH Cochin-Broca-Hôtel Dieu, APHP, Paris, France; 5 Sorbonne Paris Cité, Université Paris Descartes, Paris, France; 6 EFFI-STAT, Paris, France

**Keywords:** Clinical research, External validity, COPD exacerbations

## Abstract

**Background:**

In patients with chronic obstructive pulmonary disease, all efforts should be made to prevent exacerbations because each event modifies the trajectory of the disease. Treatment recommendations are mostly built on results from randomized controlled trials (RCTs) whose methodology ensure internal validity. However, their relevance may be compromised by the lack of generalizability, due to poor representability of study populations compared to real-life patients.

In order to delimit to whom the results of studies on current and future treatments apply, we sought to identify and characterize the fraction of COPD population that would be eligible for inclusion into RCTs aiming at decreasing exacerbation risk.

**Methods:**

We used the Initiatives-BPCO database, a French cohort of 1309 real-life COPD patients monitored in academic centers. We identified industry-sponsored phase III and IV trials that enrolled more than 500 patients, lasted at least one year and used exacerbations related endpoints. Eligibility criteria were extracted from each trial and applied to the patients.

**Results:**

The eligibility criteria of 16 RCTs were applied to the 1309 patients. The most discriminating eligibility criteria were FEV1, minimum exacerbation rate in the previous year and smoking history, responsible for the exclusion of 39.9, 36.7 and 16.8% of patients, respectively. Altogether, 2.3 to 46.7% of our patients would have satisfied all eligibility criteria.

**Conclusion:**

These analyses confirm that an important gap exists between real-life patients and clinical trials populations in COPD, which limits the relevance of results and therefore should be considered when grading levels of evidence and designing future studies.

## Background

Chronic Obstructive Pulmonary Disease (COPD) is a major public health concern worldwide due to its high prevalence, major impact on health status and elevated costs [1–3]. In most patients, the natural course of the disease is complicated by acute episodes of symptom flares called exacerbations. Frequent exacerbations, especially of the moderate and severe subtypes, have been shown to impair quality of life [4], accelerate the decline in lung function [5] and decrease survival [6].

Each exacerbation has the potential to alter the trajectory of the disease course, increasing the risk for subsequent events [7, 8] and shortening the time to the next event [6]. The decisive turning point may be the second severe episode, which delineates a new phase of the disease associated with higher mortality rates [6].

Facing this, all efforts should be made to prevent COPD exacerbations [9]. Several marketed drugs have been shown to decrease the exacerbation rate, and others are currently being tested through randomized controlled trials (RCTs) for this purpose. Treatment recommendations for the management of COPD and related exacerbations are mostly built on these RCTs results [10]. For both scientific community and public opinions, the high level of evidence produced by RCTs drives treatment recommendations and labels the clinical management as “evidence-based”.

However, despite a strong methodology ensuring internal validity of results, skepticism about the representativeness of such trials populations towards real-life patients has grown [11–18]. As a consequence of narrow eligibility criteria, efficacy results may not apply to some patients and, more importantly, safety results may underestimate potential side effects. As a result, the importance of real-life evidence to complement RCTs has been emphasized and these study designs have been positioned within a global framework of therapeutic research [19].

The GOLD document on COPD management [10] acknowledges that the lack of external validity may compromise the applicability of trials’ results to a broader population. Nonetheless it remains difficult to set apart - among the “excluded” patients - the patients who could benefit from a treatment from those who would not.

In order to delimit to whom the results of studies on current and future treatments apply, we sought to identify and characterize patients from a well-characterized real-life hospital-based COPD cohort (Initiatives BPCO) who would be eligible for inclusion into industry-sponsored RTCs on pharmacological treatments of COPD aiming at decreasing exacerbation risk.

## Methods

### Patients

We used the Initiatives-BPCO French cohort, which contained 1309 patients at the time of the present analysis. Patients in this cohort were recruited under stable condition in 18 academic centers in France. Patients recruited in this cohort were aged ≥40 years with spirometry-confirmed diagnosis of COPD, as defined by post-bronchodilator FEV1/FVC < 0.7. The exclusion criteria included a predominant diagnosis of asthma, bronchiectasis, or any other significant respiratory disease. Data collection has been extensively described previously [20]. Briefly, a case-report form was used to collect in patient’s medical file demographic data, smoking history, physician-diagnosed comorbidities, symptoms, dyspnea (modified Medical Research Council scale), results of pulmonary function tests, and the number of exacerbations and hospitalizations in the previous year. The cohort was approved by the Ethics Committee of Versailles, France (ref 04–479). All patients have provided informed consent for the anonymized recording and analysis of their data.

*Selection of RCTs and eligibility criteria.*

Using clinicaltrials.com and Pubmed websites, we selected between January 2000 and December 2016 all industry-sponsored phase III and IV RCTs that enrolled more than 500 COPD patients, lasted at least 1 year and aimed at decreasing exacerbations risk. For twin studies, we kept only one study in the analysis. All the eligibility criteria used in these trials were compiled.

### Analysis of eligibility of patients with COPD to RCTs

To determine whether patients recruited in the Initiatives-BPCO cohort would have been eligible to RCTs, our analysis first focused on the eligibility criteria per se. It allowed the identification of the most frequently required criteria. By applying criteria to the patients from the Initiatives-BPCO cohort, we were able to assess the share of patients fulfilling each criterion. We could then rank the most discriminating criteria.

**Fig. 1:**
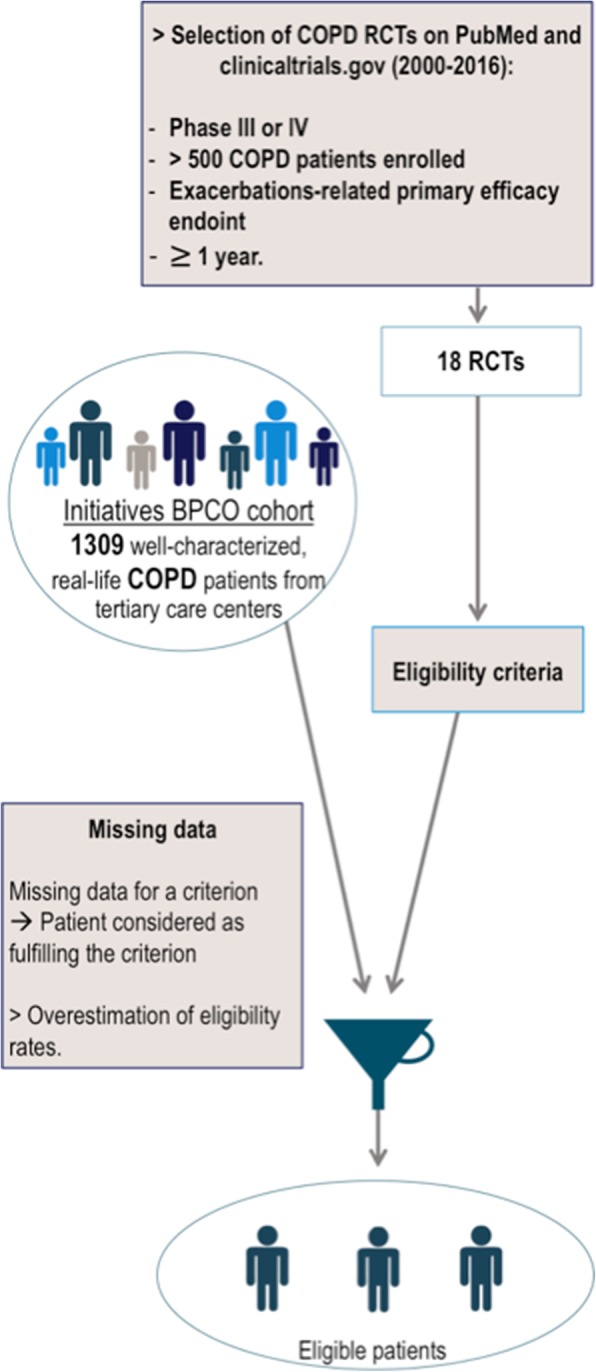
Methods used to assess the proportion of eligible patients within a real-life hospital-based cohort of COPD patients

The second analysis aimed at applying the set of criteria of each RCT to each patient from the cohort in order to assess the proportion of patients that would be eligible to join each trial (Fig. 1).

The third and final part of the study was to distinguish the eligibility rates of different groups of RCTs differing by their phases of development, pharmacological class of tested agents and starting year.

Several data were not collected or missing in the Initiatives-BPCO database. When this occurred, participants were deemed to remain eligible by the criterion we could not assess. This decision was made in order to avoid underestimating the number of patients that would have been eligible.

All analyses were performed using GraphPad Prism version 7.03 for windows (GraphPad Software, LaJolla California USA, www.graphpad.com). Phases, pharmacological agents and starting years’ comparisons were performed using Mann-Whitney test, one-way ANOVA followed by Tukey’s multiple comparison and Kruskal-Wallis tests, respectively.

## Results

### RCTs

The online search for trials provided 16 RCTs fulfilling our criteria (Table 1).

**Table 1:** RCTs selected for the analysis

Clinicaltrials number	Title	Investigationnal product	Phase	Starting year	Number of patients enrolled	Percentage of eligible patients
NCT00430729	Effect of Roflumilast on Exacerbation Rate in Patients With Chronic Obstructive Pulmonary Disease. A 52 Weeks Double Blind Study With 500mcg Roflumilast Once Daily Versus Placebo. Ratio-Study.	Roflumilast	III	2003	1100	8.8%
NCT01120691	A 64-week Treatment, Multi-center, Randomized, Double-blind, Parallel-group, Active Controlled Study to Evaluate the Effect of QVA149 (110/50 μg o.d.) vs NVA237 (50 μg o.d.) and Open-label Tiotropium (18 μg o.d.) on COPD Exacerbations in Patients With Severe to Very Severe Chronic Obstructive Pulmonary Disease (COPD)	Indacaterol/ Glycopyrronium	III	2010	2224	2.3%
NCT00909779	A Large Simple Safety Study of Arformoterol Tartrate Inhalation Solution in Subjects With Chronic Obstructive Pulmonary Disease	Arformoterol	III	2009	841	5.3%
NCT00419744	A Phase IIIB, 12-Month, Double-blind, Double-dummy, Randomised, Parallel-group, Multicentre Exacerbation Study of SYMBICORT® pMDI 160/4.5 μg × 2 Actuations Twice-daily and 80/4.5 μg × 2 Actuations Twice-daily Compared to Formoterol Turbuhaler® 4.5 μg × 2 Inhalations Twice-daily in COPD Subjects	Budesonide/ Formoterol	III	2007	1200	35.8%
NCT01009463	HZC102871: A 52-week Efficacy and Safety Study to Compare the Effect of Three Dosage Strengths of Fluticasone Furoate/Vilanterol Inhalation Powder With Vilanterol on the Annual Rate of Exacerbations in Subjects With Chronic Obstructive Pulmonary Disease (COPD)	Fluticasone / Vilanterol	III	2009	1626	19.1%
NCT01126437	A Randomized, Active-controlled, Double-blind, Double-dummy, Parallel Group Design, Multi-center Trial to Compare the Efficacy and Safety of 2.5 μg and 5 μg Tiotropium Inhalation Solution Delivered by the Respimat Inhaler With Tiotropium Inhalation Capsules 18 μg Delivered by the HandiHaler (TIOSPIR)	Tiotropium (respimat)	III	2010	17,210	46.7%
NCT01443845	Roflumilast in Chronic Obstructive Pulmonary Disease (COPD) Patients Treated With Fixed Dose Combinations of Long-acting ß2-agonist (LABA) and Inhaled Corticosteroid (ICS)	Roflumilast	IV	2011	2300	5.7%
NCT01329029	Effect of Roflumilast on Exacerbation Rate in Patients With COPD Treated With Fixed Combinations of LABA and ICS. A 52-week, Randomised Double-blind Trial With Roflumilast 500 μg Versus Placebo. The REACT Trial	Roflumilast	IV	2011	1945	5.7%
NCT00361959	A Multicentre, Randomised, Double-Blind, Double Dummy, Parallel Group, 104 Week Study to Compare the Effect of the Salmeterol/Fluticasone Propionate Combination Product (SERETIDE) 50/500mcg With Tiotropium Bromide 18 Mcg on the Rate of Exacerbations in Subjects With Severe Chronic Obstructive Pulmonary Disease (COPD)	Fluticasone/ Salmeterol	IV	2003	1270	5.0%
NCT00563381	Effect of Inhalation of Tiotropium Once Daily 18 Mcg Versus Salmeterol Twice Daily 50 Mcg on Time to First Exacerbation in COPD Patients (a Randomised, Double-blind, Double-dummy, Parallel Group, One-year Study).	Tiotropium	IV	2008	7376	27.2%
NCT00115492	A Randomized, Double-Blind, Parallel Group, 52-week Study to Compare the Effect of the Fluticasone Propionate/Salmeterol DISKUS Combination Product 250/50mcg BID with Salmeterol DISKUS 50mcg BID on the Annual Rate of Moderate/Severe Exacerbations in Subjects with Chronic Obstructive Pulmonary Disease (COPD)	Fluticasone/ Salmeterol	IV	2004	740	7.9%
NCT02138916	Randomised, Double-blind, 56 Week Placebo-controlled, Parallel Group, Multicentre, Phase 3 Study to Evaluate the Efficacy and Safety of 2 Doses of Benralizumab in Patients With Moderate to Very Severe COPD With a History of Exacerbations	Benralizumab	III	2014	1626	13.9%
NCT02579850	52-week, Double Blind, Randomized, 2 Active Parallel Arms Study of Fixed Combination CHF 5993 Administered vs Ultibro® in COPD Patients	Béclomethasone/ Formoterol/ Glycopyrronium	III	2015	1534	4.1%
NCT02465567	A Randomized, Double-Blind, Multi-Center, Parallel-Group Study to Assess the Efficacy and Safety of PT010 Relative to PT003 and PT009 on COPD Exacerbations Over a 52-Week Treatment Period in Subjects With Moderate to Very Severe COPD (Ethos)	Budesonide/ Formoterol/ Glycopyrronium	III	2015	8000	32.2%
NCT02105961	Study MEA117113: Mepolizumab vs. Placebo as Add-on Treatment for Frequently Exacerbating COPD Patients Characterized by Eosinophil Level	Mepolizumab	III	2014	660	16.3%
NCT02296138	A Randomised, Double-blind, Active-controlled Parallel Group Study to Evaluate the Effect of 52 Weeks of Once Daily Treatment of Orally Inhaled Tiotropium + Olodaterol Fixed Dose Combination Compared With Tiotropium on Chronic Obstructive Pulmonary Disease (COPD) Exacerbation in Patients With Severe to Very Severe COPD. [DYNAGITO]	Tiotropium/ Olodaterol	III	2015	7800	27.7%

**Fig. 2:**
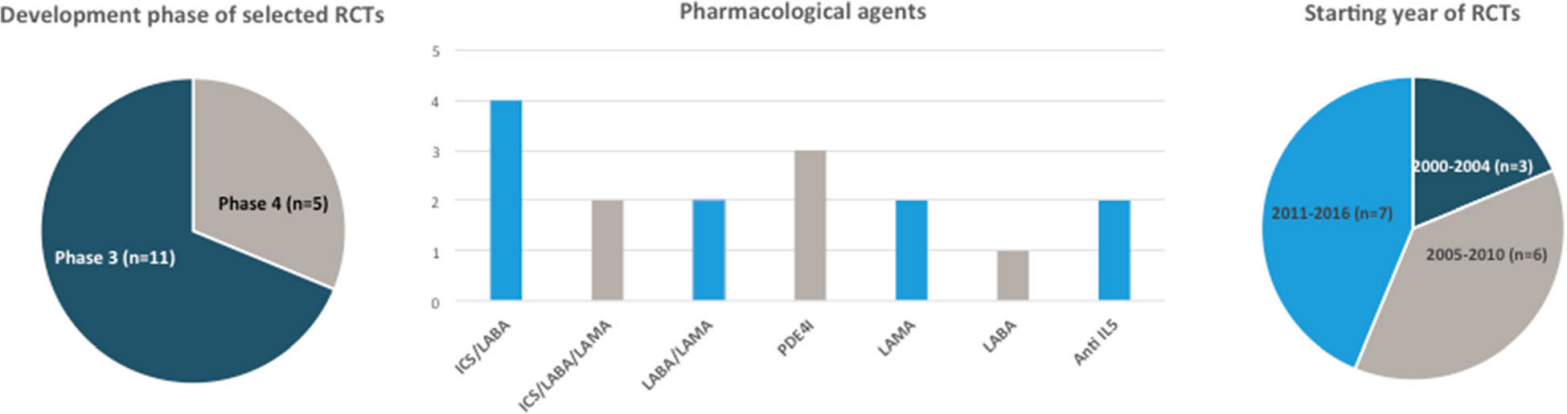
Characteristics of the RCTs selected for the analysis

From these 16 RCTs, 11 were phase III trials. The pharmacological agents tested comprised a panel consistent with the different options marketed or being developed in COPD. The distribution of trials based on their starting year covered the whole research timeframe considered for this study (Fig. 2).

### *Description of eligibility criteria* (Table 2)

**Table 2:** Eligibility criteria and related exclusion rates

	Criterion	Total number of trials setting the criterion	Cut-off values of the criterion	Number of trials setting the criterion	Availability of data in Initiative BPCO database	Percentage of patients excluded by the criterion
Demographics	Minimum age	16 (100%)	40 years	16 (100%)	yes	1.1%
	Maximum age	3 (18.8%)	80 years	2 (12.5%)	yes	5.3%
			85 years	1 (6.3%)	yes	0.8%
Disease diagnosis and severity	Smoking history	15 (93.8%)	Current or former smoker	15 (93.8%)	yes	4.9%
			At least 10 PY	11 (68.8%)	yes	17.6%
			At least 15 PY	1 (6.3%)	yes	20.2%
			At least 20 PY	2 (12.5%)	yes	25.1%
	Airway obstruction	16 (100%)	FEV1/FVC < 0,7	16 (100%)	yes	0%
	Maximum FEV1^a^	15 (93.8%)	49% (GOLD 3 threshold)	8 (50%)	yes	54.4%
			79% (GOLD 2 threshold)	1 (6.3%)	yes	9.5%
			70%	3 (18.8%)	yes	22.7%
			65%	2 (12.5%)	yes	31.2%
			60%	1 (6.3%)	yes	45%
	Minimum FEV1	1 (5.5%)	21%	2 (12.5%)	yes	8.8%
	Minimum exacerbation rate during the previous year	14 (87.5%)	1 event	10 (62.5%)	yes	36.4%
			2 events	4 (25%)	yes	62.2%
	Maximum exacerbation rate during the previous year^b^	13 (81.3%)	24 events	2 (12.5%)	yes	0%
			12 events	8 (50%)	yes	0.1%
			9 events	3 (18.8%)	yes	0.8%
Disease control	COPD symptoms	4 (25%)	Evidence of chronic productive cough	2 (12.5%)	no	Uncollected data (assumed 0%)
			mMRC score > 1	1 (6.3%)		
			CAT > 10	1 (6.3%)		
Concomitant treatments restrictions^c^	Long term oxygen therapy	7 (43.8%)	< 12 h/day	1 (6.3%)	use of oxygenotherapy assessed	11.8%
			< 15 h/day	1 (6.3%)		
			< 16 h/day	1 (6.3%)		
			Not daily	4 (25%)		
	Oral corticosteroids	6 (37.5%)	Forbidden	4 (25%)	Intake of OCS assessed	3.0%
			< 10 mg/day	2 (12.5%)		
	Previous participation in a pulmonary rehabilitation program	3 (18.8%)	Forbidden	3 (18.8%)	no	Uncollected data (assumed 0%)
Comorbidities restrictions^d^	Concomitant pulmonary diseases	15 (93.8%)	Forbidden	15 (93.8%)	Only asthma assessed	13.1%
	History of pulmonary surgery	7 (43.8%)	Lobectomy	4 (25%)	Only transplantation assessed	0%
			Lung volume reduction	6 (37.5%)		
			Transplantation	2 (12.5%)		
	History of cancer	8 (50%)	Forbidden	1 (6.3%)	no	Uncollected data (assumed 0%)
			Forbidden if within 1 year	1 (6.3%)		
			Forbidden if within 5 years	6 (37.5%)		
	Cardiovascular comorbidities (including arterial hypertension, heart failure, myocardial infarction and arrythmias)	10 (62.5%)	Forbidden	10 (62.5%)	Cardiovascular comorbidities assessed	16.1%
	Endocrine disorders	5 (31.3%)	No diabetes	2 (12.5%)	yes	13.6%
			No thyrotoxicosis	3 (18.8%)	No	Uncollected data(assumed 0%)
			No obesity (BMI > 45 kg/m2)	1 (6.3%)	No	Uncollected data(assumed 0%)
	Renal impairement	4 (22.2%)	No moderate or severe renal failure	3 (18.8%)	No	Uncollected data(assumed 0%)
	Allergic status	3 (18.8%)	No atopic status	2 (12.5%)	yes	11%
			No allergic rhinitis	3 (18.8%)	yes	21.2%
	History or current drug or alcohol abuse	8 (50%)	Forbidden	8 (504%)	No	Uncollected data(assumed 0%)
Compliance	History of poor compliance to medication prescription	3 (18.8%)	Forbidden	3 (18.8%)	No	Uncollected data(assumed 0%)
Pregnancy and breastfeeding	Pregnancy and breastfeeding	8 (50%)	Forbidden	8 (50%)	No	Uncollected data (assumed 0%)

*a. maximum* FEV1 was required to be met post bronchodilator in 13 trials, pre bronchodilator in 1 trial. Timing of measurement was not specified in 2 trials

b. None of the trials set a maximum number of exacerbations but some did not allow study participation if an event occurent 2 weeks, 4 weeks or 6 weeks before enrolment. In these trials we excluded patient who experienced more than 24, 12 or 9 events in the previous year, respectively

c. Excluding pharmacological contraindications with study drugs

d. Inclusion of patients with unstable or seriously significant disease of any kind was forbidden. Here are presented

### Analysis of eligibility rates

Within our real-life population, eligibility for inclusion into RCTs ranged from 2.3 to 46.7% of patients depending on the trial with a mean eligibility rate of 16.5% (95% CI, 9.2–23.7).

Among all trials, a mean of 39.9% of our patients would fail because of an FEV1 outside the accepted range. Insufficient exacerbation rate during the previous year would be responsible for the exclusion of 36.7% of patients. Smoking history criteria would be responsible for the exclusion of patients with a cumulative consumption lower than required (16.8% of patients). The fourth and fifth most selecting criteria would be comorbidity-related exclusion criteria: cardiovascular comorbidities and asthma restrictions leading to the ineligibility of 16.1 and 11.5% of patients respectively.

**Fig. 3:**
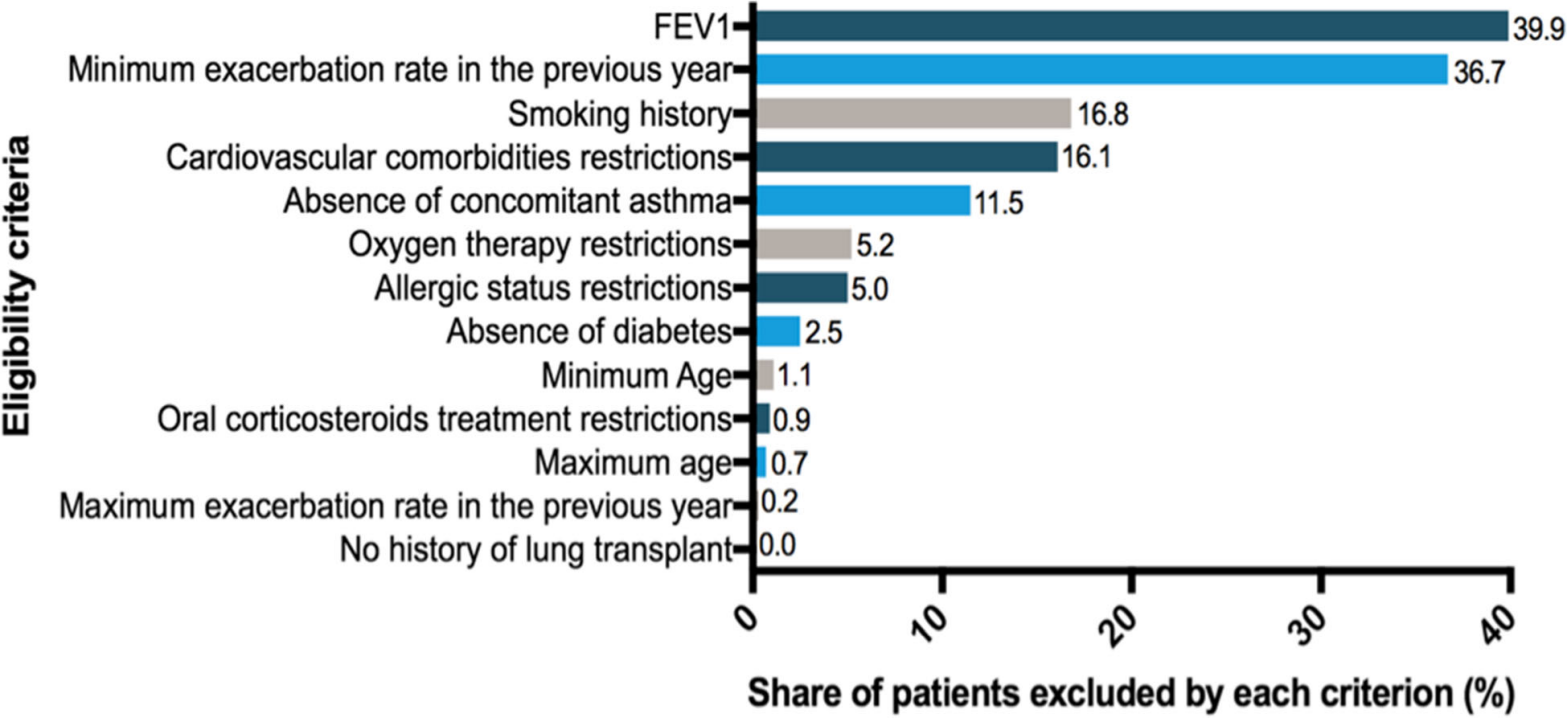
Ranking of selection criteria used in COPD Randomized Controlled Trials (mean percentage of ineligible patients in the Initiatives BPCO database due to the criterion among all RCTs)

The mean exclusion rates among the 16 trials for criteria related to oxygen therapy, allergic status, diabetes, age, oral corticosteroids intake, upper limit of exacerbations annual rate and history of lung transplant would be less than 6%. (Fig. 3).

### Eligibility rates in subgroups of trials

**Fig. 4:**
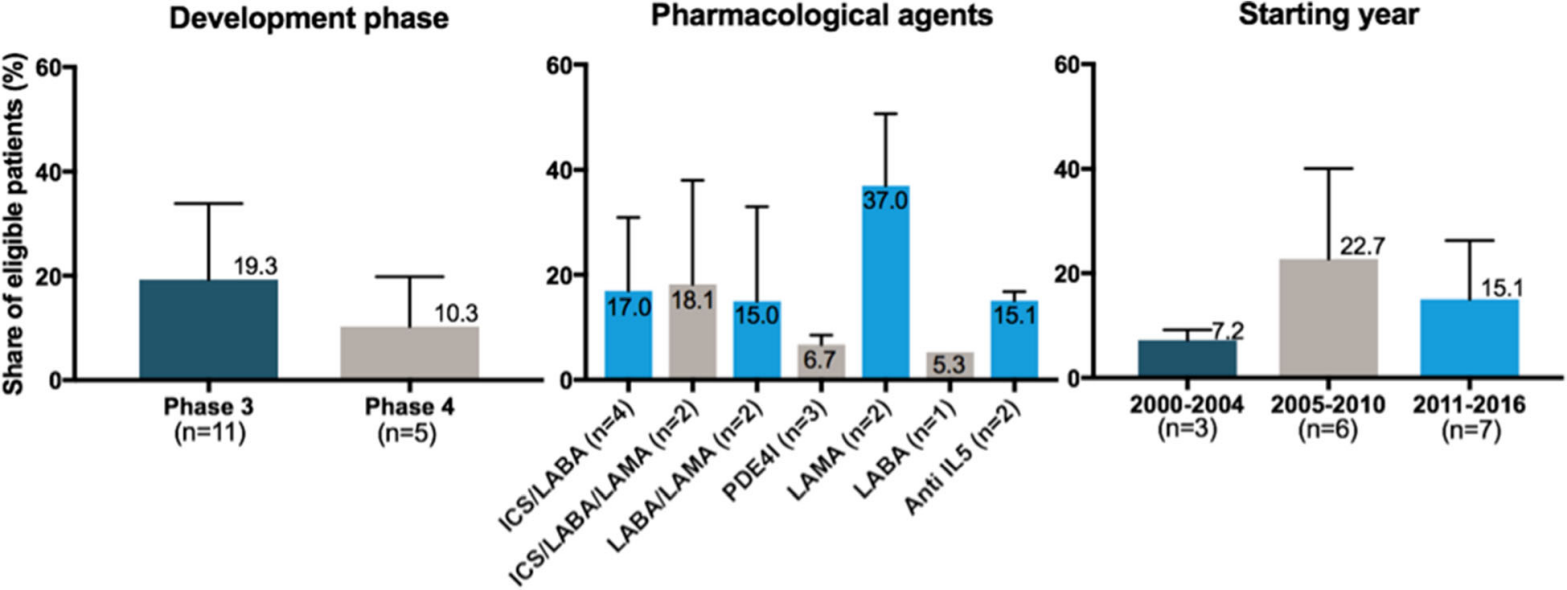
Eligibility rates in subgroups of trials differing by their development phases, pharmacological class of tested agents and starting year

Phase IV trials (*n* = 5) have a mean eligibility rate of 10.3% while phase III trials (*n* = 11) have a mean eligibility rate of 19.3%. These results are shown in Fig. 4 along with eligibility rates by pharmacological class of tested agents and starting year of RCTs.

## Discussion

In this large cohort of 1309 well-characterized and followed-up COPD patients from French centers, a majority of patients would not be eligible for enrolment into RCTs assessing the efficacy of pharmaceutical intervention for COPD exacerbations. Importantly, our study described precisely the main reasons for ineligibility in these real-life patients with COPD and illustrate their evolution over 16 years. These data may contribute to the design of future RCTs with the aim of limiting exclusion of patients.

One limitation is the use of an academic cohort as the “real-life” reference. Moreover, enrollment in our cohort is itself limited by some eligibility criteria. We focused on the trials aiming at decreasing the exacerbation risk because they require the enrolment of patients experiencing severe exacerbations, the patients observed from our cohort being closer to the target population.

We must however acknowledge the potential gap between our cohort and real-life patients. Thus, our eligibility rates may be overestimated but this will not bias our conclusions.

Our findings of eligibility rates ranging from 2.3 to 46.7% with a mean of 16.5% are consistent with previously published results: we identified 6 studies dealing with external validity of COPD randomized controlled trials over the last two decades [11–16]. None of them focused specifically on trials aiming at decreasing exacerbation rates but our results are clearly complementary with theirs. Herland applied a limited set of criteria from a fictive “typical RCT” to 366 patients [14]. They found a share of 17.2% of potentially eligible patients. These results are consistent with those obtained by Kruis (17 to 42% out of 3508 patients from 7 European primary care databases would have been eligible to enter 6 large industrial RCTs [15]) and our own results. Halpin found eligibility rates ranging from 3.5 to 57.6% of 36,893 patients from primary and secondary care [11].

Scichilone and Travers both focused on RCTs supporting the old GOLD guidelines for management of COPD in a hospital cohort [12] and in general population [13]. Eligibility rates of 16.9% and 0 to 9% respectively show worryingly the impact of the lack of representativity on the recommendations of patients management at the collective level. These results point the poor external validity of many RCTs.

One other limitation is the existence of uncollected data. In this case, a patient was considered fulfilling the missing criterion. This could have resulted in overestimating the eligibility rates. However, with higher eligibility rates than actual rates, our conclusions would even be strengthened. Moreover, we only considered eligibility criteria at inclusion, some run-in or randomization criteria may apply.

Decreasing the number of exacerbations (especially the severe ones) is a major challenge in COPD. Promising results obtained in RCTs often contrast with the modest results observed in daily practice. The evidence-based management of individual patients relies on a meticulous analysis of external validity of RCTs results: Would my patient have been includable in this trial? If not, can I extrapolate the results to my patient considering the unmet criteria?

Some eligibility criteria may have a limited impact on external validity: excluding patients who previously participated in rehabilitation program for example may not exclude a specific sub-population.

Some other criteria are selecting but relevant with regard to the primary endpoint. We focused on RCTs aiming at decreasing the exacerbation risk, which makes it appropriate to enroll patients with a history of exacerbation(s). We must however mention that considering the study primary efficacy endpoint is crucial in the extrapolation of results to a patient. Rothnie and colleagues recently show that almost 52% of COPD patients do not exacerbate in the first year of follow up and that a 26% do not exacerbate during a 10 years follow up. Treating these patients with medications that only prove their efficacy on exacerbation rate would not be relevant, but it remains difficult if not impossible to predict the risk of exacerbation accurately on an individual basis [21].

In contrast, some other eligibility criteria are responsible for the exclusion of important sub-populations of patients whose COPD exacerbations triggers and pathophysiology may differ from the ones of enrolled patients.

To illustrate this point, three groups of criteria require particular attention:

First, a selective FEV1-related criterion is set in 94% of RCTs and would be responsible for the exclusion of 39.9% of patients from our database. Only one trial allowed the enrollment of patients with mild airflow limitation. To assess medications aiming at decreasing exacerbation risk, most RCTs only allowed the enrollment of patients with severe airflow obstruction, which is not necessarily correlated to exacerbation risk. Another interesting finding of Kruis is the proportion of frequent exacerbators among GOLD 1 patients: 12% are frequent exacerbators with ≥ 2 events a year. 34% have at least 1 event a year. Woodruff has similar findings pointing that COPD exacerbations and symptoms may occur in patient with preserved pulmonary function [22]. We cannot assume that RCTs results are extrapolatable to these patients.

The second set of criteria is related to the potential risk factors for COPD. In 94% of RCTs in our analysis, only current or former smokers were enrolled. There is no doubt as to the responsibility of tobacco as a risk factor in COPD. However, it is reductive to consider only this risk factor in drug development as COPD may develop in never smokers (up to 27% in some studies) [23–25].

Similarly, wheezing and a past history of asthma have been shown to be major risk factors for COPD exacerbations [8, 26]. These patients are excluded from 87% of RCTs in our study. Thus, patients with an Asthma-COPD overlap are not considered in COPD studies. They are also excluded from asthma studies [18] which makes it difficult to define their management in an evidence-based manner.

Because patients are excluded from RCTs based on risk factors, the pharmaceutical industry does not consider the heterogeneity of patients with COPD in the development of medications [27]. As a correlate, this aspect is poorly considered either during the registration process by regulatory authorities.

Pathophysiological mechanisms may differ and we cannot assume that RCTs results apply to COPD phenotypes in which they have not been tested.

Lastly, the set of exclusion criteria related to comorbidities raises additional concerns about the applicability of patients’ outcomes in RCTs to real-life situations. Cardiovascular comorbidities as well as atopy and diabetes are common barriers to inclusion. These are also highly prevalent in COPD patients, associated to worse outcomes and to different response to therapy. If allergic status and diabetes rates are consistent with previous epidemiologic studies, we found lower cardiovascular comorbidities proportions in our cohort than previously reported [28, 29] while these where not exclusion criteria to enter the initiatives-BPCO cohort. Only arterial hypertension, heart failure, myocardial infarction and arrhythmias were collected in our database.

Several comorbidities have been shown to be associated with the “frequent exacerbator” phenotype and to increase the exacerbation risk [30]. This is particularly the case for heart failure, asthma and diabetes, which are all frequent exclusion criteria in RCTs in our study. Excluding patients with higher exacerbation risk is then questionable in studies aiming at decreasing exacerbation risk.

All these restrictions in RCTs lead to poor external validity. It is common belief to think that this gap between trials and real-life populations exists more in phase III than in phase IV trials. We found that phase 4 are also poorly representative.

We could then hypothesize that meta-analyses could help increasing the applicability of results to patients excluded in some but not all studies. However, our results highlight important similarities of eligibility criteria among RCTs. Consequently, tested populations are very homogeneous and meta-analyses cannot fully bridge the gap.

These results emphasize the need for high quality real-life therapeutic research to complement RCTs and determine whether, to which extent and in whom efficacy results translate into real-life effectiveness [31].

## Conclusion

Our study shows that a majority of real-life COPD patients are not eligible for inclusion in RCTs assessing the effects of therapeutic intervention on COPD exacerbations. The excluded populations may present distinct triggers for exacerbations and we cannot assume the applicability of study results to these patients.

This important gap between real-life patients and clinical trials populations limits the external validity of RCTs and therefore should be considered when grading levels of evidence and designing future studies to ensure evidence-based medical decision-making.

## Data Availability

The datasets used and/or analysed during the current study are available from the corresponding author on reasonable request after approval from initiatives BPCO scientific committee.

## Abbreviations

BMI: Body Mass Index
CAT: COPD Assessment Test
COPD: Chronic Obstructive Pulmonary Disease
FEV1: Forced Expiratory Volume in 1 s
FVC: Forced Vital Capacity
GOLD: Global initiative for chronic Obstructive Lung Disease
ICS: Inhaled Corticosteroids
IL5: Interleukin 5
LABA: Long Acting Beta2 Agonist
LAMA: Long Acting Muscarinic Antagonist
Mcg: Microgram
mMRC: Modified Medical Research Council
PE4i: Phosphodiesterase type 4 Inhibitor
PY: Pack Year
RCT: Randomized Controlled Trial
